# ZuCo, a simultaneous EEG and eye-tracking resource for natural sentence reading

**DOI:** 10.1038/sdata.2018.291

**Published:** 2018-12-11

**Authors:** Nora Hollenstein, Jonathan Rotsztejn, Marius Troendle, Andreas Pedroni, Ce Zhang, Nicolas Langer

**Affiliations:** 1 Department of Computer Science, ETH Zurich, Zurich, Switzerland; 2 Methods of Plasticity Research, Department of Psychology, University of Zurich, Zurich, Switzerland; 3 University Research Priority Program (URPP) Dynamics of Healthy Aging, Zurich, Switzerland; 4 Neuroscience Center Zurich (ZNZ), Zurich, Switzerland

**Keywords:** Computer science, Language, Reading

## Abstract

We present the *Zurich Cognitive Language Processing Corpus* (ZuCo), a dataset combining electroencephalography (EEG) and eye-tracking recordings from subjects reading natural sentences. ZuCo includes high-density EEG and eye-tracking data of 12 healthy adult native English speakers, each reading natural English text for 4–6 hours. The recordings span two normal reading tasks and one task-specific reading task, resulting in a dataset that encompasses EEG and eye-tracking data of 21,629 words in 1107 sentences and 154,173 fixations. We believe that this dataset represents a valuable resource for natural language processing (NLP). The EEG and eye-tracking signals lend themselves to train improved machine-learning models for various tasks, in particular for information extraction tasks such as entity and relation extraction and sentiment analysis. Moreover, this dataset is useful for advancing research into the human reading and language understanding process at the level of brain activity and eye-movement.

## Background & Summary

Natural language processing (NLP), a fundamental aspect of artificial intelligence, aims at teaching computers to process features of natural language data, such as the sentiment of a sentence or relational information between text entities. Due to the advances in AI in the recent years, NLP applications have greatly improved their performance in automatically analyzing and extracting knowledge from text and speech.

However, in order to train NLP applications, large labeled datasets are often required. For instance, to train a sentiment analysis system, which predicts the sentiment of a sentence (i.e. positive/negative/neutral), thousands of annotated sentences are needed. Typically, human annotators must read these training sentences and assign a sentiment to each one. Clearly, this reflects a significant investment. Our long-term goal is to replace this labor-intensive and expensive task with physiological activity data recorded from humans while reading sentences. That is to say, we aim to find and extract relevant aspects of text understanding and annotation directly from the source, i.e. eye-tracking and brain activity signals during reading. By way of illustration, opinions and sentiments are elicited from a person reading text, which is reflected in their brain activity. Hence, it should be possible to decode this information from the recorded brain activity data with machine learning techniques and bypass - or at least complement - manual human annotation.

Whether it is possible to decode such information from brain activity is an empirical question and has not been answered so far. Yet, previous studies have demonstrated that eye movement information improves NLP tasks such as part-of-speech tagging^1^, sentiment analysis^2^ and word embedding evaluation^3^. In addition, there are some available resources of subjects’ eye-movement recordings while reading text (e.g. the Dundee corpus^4^ and the GECO corpus^5^). However, while there are studies that combine EEG and eye-tracking from a psycholinguistic motivation (e.g. response to syntactically incorrect sentences^6^), up to now there is no dataset available that combines eye-tracking and brain activity that is tailored for training machine learning algorithms to perform NLP tasks.

In this article we present a dataset of simultaneous electroencephalography (EEG) and eye-tracking data of 12 subjects reading natural sentences. We therefore provide a dataset that will enable researchers to advance the training of NLP applications using rich physiological data. Preliminary experiments with positive results^7,8^ show the potential capability of applying this data successfully in NLP.

The study design includes three tasks: two normal reading paradigms, which differ in the text materials, and one task-specific paradigm, where subjects had to actively engage in a language comprehension exercise. In all tasks, the text understanding was tested by specific questions. Table 1 shows a detailed schematic overview of the tasks. The reading materials recorded for the Zurich Cognitive Language Processing Corpus (ZuCo) contain sentences from movie reviews from the *Stanford Sentiment Treebank*^9^ and biographical sentences about notable people from the *Wikipedia relation extraction corpus*^10^. In addition, all subjects completed a standardized test to assess their vocabulary and language proficiency (Lexical Test for Advanced Learners of English)^11^.

A prominent feature of this dataset is the personal reading speed. The sentences were presented to the subjects in a naturalistic reading scenario, where the complete sentence is presented on the screen and the subjects read each sentence at their own speed, i.e. the reader determines him/herself for how long each word is fixated and which word to fixate next.

Brain-electric correlates of reading have traditionally been studied with word-by-word presentation, a condition that eliminates important aspects of the normal reading process and precludes direct comparisons between neural activity and oculomotor behavior^12^. Thus, it is important to emphasize the value of the simultaneous EEG and eye-tracking recordings of this study. Eye-tracking permits us to define exact word boundaries in the timeline of a subject reading a sentence, allowing to extract EEG signals for each word.

We want to highlight the re-use potential of this data. It allows to conduct experiments for different NLP tasks. Possible NLP applications are information extraction for text mining, including entity and relation discovery, and semantic tasks, such as sentiment analysis. To train machine learning system the number of samples (i.e. words and sentences) is crucial. Hence, in this work we focused more on the number of sentences recorded than the number of subjects. While this dataset has been created with machine learning and natural language processing as its primary application, this data can also be used to analyze the human reading process from a neuroscience perspective. It can be used for linguistic and (neuro-)psychological studies to generate new hypotheses (exploratory analyses), but these hypotheses should then be tested on a higher number of subjects to account for the variability of reading strategies across subjects. The technical validation of this dataset, described further below, is proof of the quality of the recordings.

## Methods

### Participants

Data were recorded from 12 healthy adults, all native English speakers (originating from Canada, USA, UK or Australia). The study included 5 female and 7 male subjects, all right-handed, of ages between 22 and 54 years. Details about the participants’ age and gender can be found in Table 2. All participants gave written consent for their participation and the re-use of the data prior to the start of the experiments. The study was approved by the Ethics Commission of the University of Zurich.

### Linguistic assessment

Vocabulary and language proficiency of the participants was tested with the LexTALE test (Lexical Test for Advanced Learners of English^11^), an unspeeded lexical decision task, which is for intermediate to highly proficient language users. The average score of all subjects on the LexTALE test was 94.69%. Additionally, Table 2 shows the detailed scores per subject. These scores are the percentages of correctly answered control questions in the respective task.

### Task overview

In the next section we present first the materials read by the subjects during the recording sessions, followed by a detailed description of each task and the experimental procedure.

### Materials

The dataset we present is composed of sentences from the *Stanford Sentiment Treebank*^9^ and the *Wikipedia relation extraction corpus*^10^.

The *Stanford Sentiment Treebank* contains single sentences extracted from movie reviews with manually annotated sentiment labels. We randomly selected 400 very positive, very negative or neutral sentences (4% of the full treebank). The 400 selected sentences are comprised of 123 *neutral*, 137 *negative* and 140 *positive* sentences.

The *Wikipedia relation extraction dataset* contains paragraphs about famous people, which were labeled with relation types. For the normal reading we randomly selected 650 sentences that contain a relation (14% of the full dataset). For the task-specific relation extraction reading we selected approximately 40 sentences of each of the following relation types: *award, education, employer, founder, job_title, nationality, political_affiliation, visited* and *wife*. Of these sentences, 300 were used in the normal reading tasks and 407 in the task-specific task (see Table 3). Note that 48 sentences are duplicates and appear in both tasks. These duplicate sentences can be found in a separate file in the online repository (Data Citation 1).

Table 3 presents the overall descriptive statistics of the materials split by task. Sentences from both parts of the dataset were presented to the subjects in three different tasks of normal reading and task-specific reading. All sentences can be also found in the MATLAB files and in separate text files (Data Citation 1), which include the sentiment and relation labels.

### Stimuli & Experimental Design

During all three tasks, the sentences were presented one at a time at the same position on the screen. Text was presented in black with font size 20-point Arial on a light grey background resulting in a letter height of 0.8 mm or 0.674°. The lines were triple-spaced, and the words double-spaced. A maximum of 80 letters or 13 words were presented per line in all three tasks. Long sentences spanned multiple lines. A maximum of 7 lines for Task 1, 5 lines for Task 2 and 7 lines for Task 3 were presented simultaneously on the screen.

**Figure 1:**
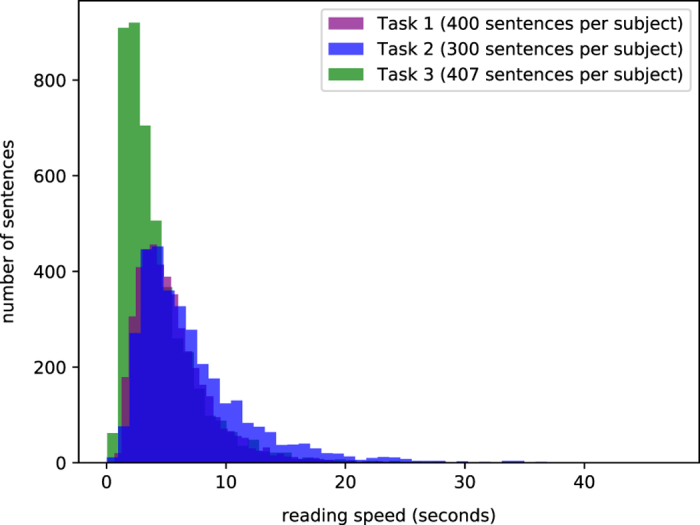
Histogram of the reading speeds of all sentences for all three tasks.

In all three tasks the subjects used a control pad to switch to the next sentence and to answer the control questions, which allowed for natural reading. Compared to RSVP (Rapid Serial Visual Representation), where each word is presented separately at an equal speed, the normal reading approach is closer to a natural reading scenario6: The subjects read each sentence at their own speed, i.e. the reader determines him/herself for how long each word is fixated and which word to fixate next. This allows for varying reading speed between the subjects; each subject reads at his/her own personal pace. Table 2 shows the average reading speed, i.e. the average number of seconds a subject spends per sentence, reported for each task. Note that a Wilcoxon test revealed significant difference in reading speeds between Task 3 and Task 2 (Z = −3.0594; p ≤ 0.01; see Fig. 1 for the distribution of the reading speeds per task). The individual reading speeds for every subject were considerably lower in Task 3 than in Task 2, since the tasks itself were different. Although the reading material was of the same type, in Task 2 passive reading was recorded, while in Task 3 the subjects had to search for a specific relation type. Thus, the task-specific reading lead to shorter passes because the goal was merely to recognize a relation in the text, but not necessarily to process the whole meaning of the sentences. The task instructions are described in detail below.

### Task 1: Normal reading (Sentiment)

#### Task overview

In the first task, the subjects were presented with positive, negative or neutral sentences for normal reading to analyze the elicitation of emotions and opinions during reading. As a control condition, the subjects had to rate the quality of the described movies in 47 of the 400 sentences. The average response accuracy compared to the original labels of the *Stanford Sentiment Treebank* is 79.53% and the response accuracy per subject can be found in Table 2 in column “Score Task 1”.

#### Stimuli & experimental design

**Figure 2:**
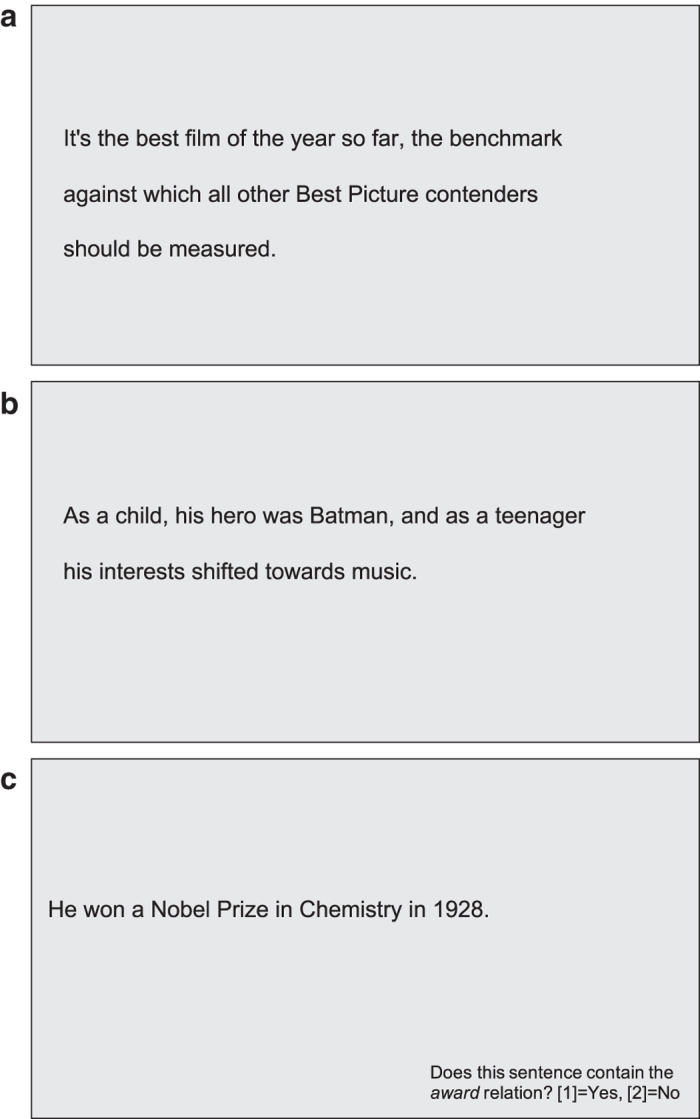
Sample screens for a sentence of each task. (**a**) Task 1 (Sentiment). (**b**) Task 2 (Normal Reading). (**c**) Task 3 (Task-specific Reading).

Figure 2a shows a sample sentence and how it was presented on the screen. The movie ratings in the control condition questions were answered with the numbers 1–5 (very bad - very good) on the control pad.

#### Participant instructions

The task was explained to the subject orally, followed by instructions on the screen:

“Please read the following sentences. After you read each sentence, press 1 to continue. Press 6 to start the task.” “Based on the previous sentence, how would you rate this movie? (very bad) |1- 2- 3 - 4 - 5 | (very good) Please press the corresponding number on the keyboard.”

### Task 2: Normal reading (Wikipedia)

#### Task overview

In the second task, the subjects were presented with sentences that contained semantic relations. The control condition for this task consisted of multiple-choice questions about the content of the previous sentence (68 sentences were followed by a question). The average response accuracy is 87.96% and the response accuracy per subject can be found in Table 2 in column “Score Task 2”.

#### Stimuli & experimental design

The sentences were presented to the subject in the same manner as in Task 1. The numbers on the control pad were used to choose the answers of the control questions. Figure 2b shows an exemplary sentence as it appeared on the screen.

#### Participant instructions

The task was explained to the subjects orally, followed by instructions on the screen:

“Please read the following sentences. After you read each sentence, press 1 to continue. Press 6 to start the task.”;

“Who was his childhood hero?

1. Superman
2. Spiderman
3. Batman

Please press the corresponding number on the keyboard.”

### Task 3: Task-specific reading (Wikipedia)

#### Task overview

In a subsequent session, the subjects were presented with similar sentences as in the second task, but with specific instructions to focus on a certain relation type. As described above, the following relation types were contained in the sentences: *award, education, employer, founder, job_title, nationality, political_affiliation, visited* and *wife*. This allows us to compare the EEG and eye-tracking signals during normal reading to reading with a specific relation in mind. As a control condition, the subjects had to report for each sentence whether a specific relation was present in the sentence or not. The relation was not present in 72 sentences. The average response accuracy on this control condition is 93.16% and the response accuracy per subject can be found in Table 2 in column “Score Task 3”.

The sentences were presented in blocks of the same relations, so the subjects would know which relation to look for without having to read the questions. Each of these blocks had a separate practice round with a definition of the relations and three sample sentences.

#### Stimuli & experimental design

Figure 2c shows a sample sentence on a screen for this specific task. Note that the control question in the bottom right was presented for each sentence.

#### Participant instructions

The task was explained to the subjects orally, followed by instructions on the screen.

Definition of the relation type of the current block, shown before the practice round:

“AWARD; While reading the following sentences please watch out for the relation between a person or their work and the award they/it received or were nominated for.”

#### Task instructions

“Please read the following sentences. After you read each sentence, answer the question below. Press 6 when you are ready.”;

“Does this sentence contain the *award* relation? [1] = Yes, [2] = No”

### Procedure

Each participant read the entire reading material in two sessions of 2–3 hours each (at the same time of day). In the first session the participants completed Task 2, followed by the first half of Task 1. In the second session the participants completed Task 3, followed by the second half of Task 1. The sentences were presented to all subjects in identical order. Before each task, a practice round of 3–5 sentences was displayed for the subject to get familiar with the task. The eye-tracking device was re-calibrated in blocks of 60 sentences (approx. every 10–15 minutes) for the first two tasks of normal reading and after every 40 sentences in the third task.

### Data acquisition

Data acquisition took place in a sound-attenuated and dark experiment Faraday recording cage. Participants were seated at a distance of 68 cm from a 24-inch monitor (ASUS ROG, Swift PG248Q, display dimensions 531.4 × 298.9 mm, resolution 800 × 600 pixels (resulting in a display: 400 × 298.9 mm), vertical refresh rate of 100 Hz). A stable head position was ensured via a chin rest. Subjects were instructed to stay as still as possible during the tasks. Participants were also offered snacks and water during the breaks and were encouraged to rest.

The experiment was programmed in MATLAB 2016b^13^, using the PsychToolbox extension^14,15^. The order of the reading paradigms was the same for all participants. Instructions for the tasks were presented on the computer screen. Participants completed the tasks sitting alone in the room, while two of the authors were monitoring their progress in the adjoining room.

### Eye-tracking acquisition

During all of the EEG paradigms, eye position and pupil size were recorded with an infrared video-based eye tracker (EyeLink 1000 Plus, SR Research, http://www.sr-research.com/) at a sampling rate of 500 Hz and an instrumental spatial resolution of <0.01°. The eye tracker was calibrated with a 9-point grid before each paradigm. Specifically, participants were asked to direct their gaze in turn to a dot presented at each of nine locations in a random order. In a validation step, the calibration was repeated until the error between two measurements at any point was less than 0.5°, or the average error for all points was less than 1°.

### Eye-tracking preprocessing and feature extraction

**Figure 3:**
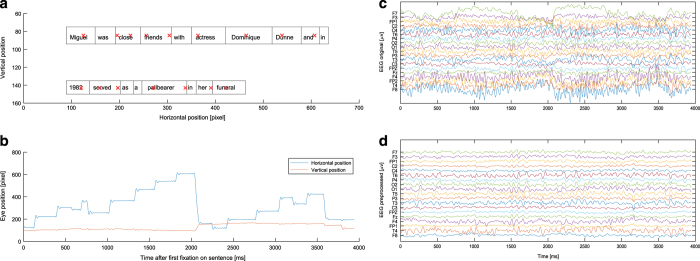
Visualization of single trial EEG and eye-tracking data. (**a**) Prototypical single sentence fixation data for a representative subject. Red crosses indicate fixations. Boxes around the words indicate the area in which fixations are allocated to the specific word. (**b**) Raw gaze data of the fixation data plotted above. (**c**) Subset of the raw EEG data during the sentence. Electrodes matching the 10–20 systems were chosen and for plotting purposes data were bandpass-filtered (0.5–30 Hz.). (**d**) Same data as in (**c**) after preprocessing.

The EyeLink 1000 tracker processes eye-position data, identifying saccades, fixations and blinks. Saccades are detected by the velocity and acceleration of the eye movements. Here, SR-research default system parameters have been used to define saccades: an acceleration threshold of 8000° per sec2, a velocity threshold of 30° per sec, and a deflection threshold of 0.1°. Fixations were defined as time periods without saccades. The dataset therefore consists of (x,y) gaze location entries for individual fixations (Fig. 3). Coordinates were given in pixels with respect to the monitor coordinates (the upper left corner of the screen was (0,0) and down/right was positive). We also provide raw sample data that can be used to validate fixation detection settings. Further, a blink can be regarded as a special case of a fixation, where the pupil diameter is either zero or outside a dynamically computed valid pupil, or the horizontal and vertical gaze positions are zero.

For later analysis, only fixations within the boundaries of each displayed word have been extracted. On the x-axis, the word boundaries were extended so that they were adjacent (Fig. 3). A Gaussian mixture model was trained on (y-axis) gaze data for each sentence to improve allocation of the eye fixations to the corresponding text lines. The number of text lines determined the number of Gaussians to be fitted within the model. Subsequently, each gaze data point was clustered to the matching gaussian and the data were realigned. As a result, each gaze data point is clearly assigned to a specific text line. Data points distinctly not associated with reading (minimum distance of 50 pixels to the text) were excluded.

On the basis of a previous eye-tracking corpus^5^ we have extracted the following eye-tracking features in MATLAB (code available in the data repository (Data Citation 1)): (I) gaze duration (GD), the sum of all fixations on the current word in the first-pass reading before the eye moves out of the word; (II) total reading time (TRT), the sum of all fixation durations on the current word, including regressions; (III) first fixation duration (FFD), the duration of the first fixation on the prevailing word; (IV) single fixation duration (SFD), the duration of the first and only fixation on the current word; and (V) go-past time (GPT), the sum of all fixations prior to progressing to the right of the current word, including regressions to previous words that originated from the current word. For each of these eye-tracking features we have additionally computed the pupil size. Furthermore, we have extracted the number of fixations and mean pupil size for each word and sentence.

Fixations that were shorter than 100 ms were excluded from the analyses, because these are unlikely to reflect fixations relevant for reading^16^, however, the raw eye-tracking data are available to assess further potential eye-tracking features.

### EEG acquisition

High-density EEG data were recorded at a sampling rate of 500 Hz with a bandpass of 0.1 to 100 Hz, using a 128-channel EEG Geodesic Hydrocel system (Electrical Geodesics, Eugene, Oregon). The recording reference was at Cz. For each participant, head circumference was measured, and an appropriately sized EEG net was selected. The impedance of each electrode was checked prior to recording, to ensure good contact, and was kept below 40 kOhm. Electrode impedance levels were checked after every third block of 60 sentences (approx. every 30 mins) and reduced if necessary.

### EEG preprocessing steps and feature extraction

The data shared in this project are available as raw data, but also preprocessed with Automagic (version: 1.4.6). The MATLAB code for the preprocessing can be found at https://github.com/methlabUZH/automagic. The data from each paradigm are saved as a separate file for each participant. In the first step of preprocessing, EEG data were imported in MATLAB (*pop_readegi.m*) and the triggers and latencies for each paradigm were extracted. One hundred and five EEG channels were used for scalp recordings and nine EOG channels were used for artifact removal. The rest of the channels lying mainly on the neck and face were discarded before data analysis.

Bad electrodes were identified and replaced. Identification of bad electrodes was based on the EEGLab plugin *clean_rawdata* (http://sccn.ucsd.edu/wiki/Plugin_list_process). This plugin removes flatline channels, low-frequency and noisy channels. A channel was defined as a bad electrode when recorded data from that electrode were correlated at less than 0.85 to an estimate based on other channels (channel criterion). Furthermore, a channel was defined as a bad channel if it had more line noise relative to its signal compared to all other channels (4 standard deviations). Finally, if a channel had a longer flatline than 5 s, it was considered as bad.

In a next step the EEG data were high-pass filtered at 0.5 and notch filtered (49–51 Hz) with a Hamming windowed-sync finite impulse response zero-phase filter (EEGLAB function *pop_eegfiltnew.m*). The filter order was defined to be 25% of the lower passband edge. Eye artifacts were removed by linearly regressing the EOG channels from the scalp EEG channels^17^. The EOG electrodes were placed on the participant’s forehead, outer and inner canthi (#'s 8, 14, 17, 21, 25, 125, 126, 127, and 128 from the HydroCel Geodesic Sensor Net). In this study, MARA^18^ (Multiple Artifact Rejection Algorithm), a supervised machine learning algorithm that evaluates ICA components, is used for automatic artifact rejection. MARA has been trained on manual component classifications, and so captures the wide range of artifacts that manual rejection detects. MARA has proven especially effective at detecting and removing eye and muscle artifact components. Specifically, MARA evaluates each component on the six algorithm features: Current Density Norm and Range Within Pattern, Fit Error k, 8–13 Hz, and Mean Local Skewness as the feature set. MARA rejects any components with artifact probabilities greater than 0.52. Subsequently, bad electrodes were interpolated by using a using spherical spline interpolation *eeg_interp.m*. Moreover, after automatic scanning, noisy channels were selected by visual inspection and interpolated. The effect of preprocessing is displayed in Fig. 3 for representative subject and sentence.

After preprocessing, the EEG and eye-tracking data were synchronized using “EYE EEG extension”^19^ to enable EEG analyses time-locked to the onsets of fixations. The synchronization is performed by identifying “shared” events and fitting a linear function to the shared event latencies in order to refine the estimation of the latency of the start- and end-event. Synchronization quality was ensured by comparing the trigger latencies recorded in the EEG and eye-tracker data. All synchronization errors did not exceed one sample (2 ms).

For the purposes of the current project, we were interested in oscillatory power in different frequency bands; however, the time-series data are shared as well. To compute oscillatory power measures, we band-pass filtered the continuous EEG signals across an entire task period for five different frequency bands resulting in a time-series for each frequency band. The independent frequency bands were determined following: *theta1* (4–6 Hz), *theta2* (6.5–8 Hz) *alpha1* (8.5–10 Hz), *alpha2* (10.5–13 Hz), *beta1* (13.5–18 Hz) *beta2* (18.5–30 Hz) and *gamma1* (30.5–40 Hz) and *gamma2* (40–49.5 Hz). We then applied a Hilbert transform to each of these time-series, resulting in a complex time-series. The Hilbert phase and amplitude estimation method yields results equivalent to sliding window FFT and wavelet approaches^20^. We specifically chose the Hilbert transformation to maintain temporal information for the amplitude of the frequency bands to enable the power of the different frequencies for time segments defined through fixations from the eye-tracking recording. Thus, for each eye-tracking feature we computed the corresponding EEG feature in each frequency band. Furthermore, we have extracted EEG features based on sentence-level by calculating the power in each frequency band. For all EEG features, we have additionally calculated the difference of the power spectra between frontal left and right homologue electrodes pairs (see the readme file for the exact electrodes). For each EEG eye-tracking feature, all channels were subject to an artifact rejection criterion of ±90μV to exclude trials with transient noise.

### Code availability

The code for the preprocessing can be found here: https://github.com/methlabUZH/automagic.

## Data Records

### Data privacy

All data are de-identified and participants gave permission for their data to be openly shared as part of the informed consent process.

### Distribution for use

Raw and preprocessed EEG and eye-tracking data are available online (Data Citation 1). EEG and eye-tracking data are available openly, along with basic personal data (age, gender, handedness) and linguistic performance measures (LexTale scores). Public data are distributed under the the Creative Commons Attribution 4.0 International Public License (https://creativecommons.org/licenses/by/4.0/).

### EEG and eye-tracking data organization

The data are stored in folders by task (Data Citation 1). Combined EEG and eye-tracking data can be found in the MATLAB files, one file per subject.

## Technical Validation

### Eye-tracking

#### Omission rates & skipping proportions

**Figure 4:**
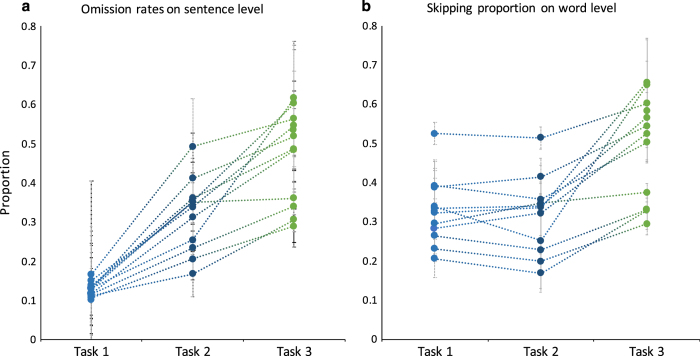
Omission rates and skipping proportions (means and standard errors) for all tasks and subjects (means and standard error). (**a**) The omission rates for each task and for each subject, where the y-axis shows the proportion of words being skipped in a sentence (0–1). (**b**) The skipping proportion (y-axis) for each task and for each subject.

**Figure 5:**
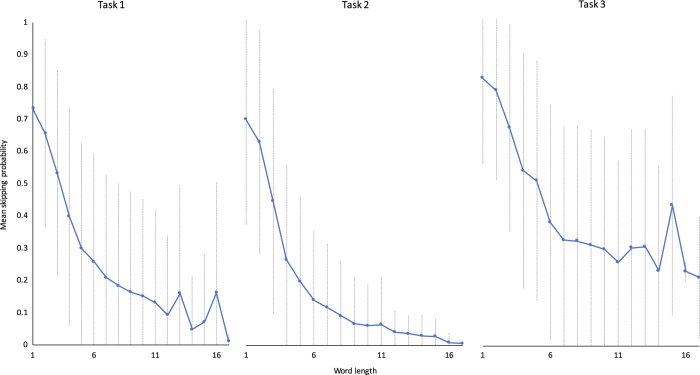
Effect of word length on the skipping proportion per task (mean and standard deviation), x-axis = word length, y-axis = mean skipping proportion.

The eye-tracking data were evaluated by analyzing the fixations made by each subject. On the one hand, we analyze the fixations on sentence level using the omission rate. The omission rate is defined as the percentage of words that is not fixated in sentence. Figure 4a shows the omission rates per task for each subject. On the other hand, we analyze the skipping proportion on the word level. The skipping proportion is the rate of words being skipped (i.e. not being fixated). Figure 4b presents the skipping proportion for all tasks and each subject. The values of the reported metrics are in accordance with values reported in other eye-tracking reading studies^5,21^. Moreover, we present the effect of word length on skipping proportion, as it has been presented previously^5^. As mentioned above, both the omission rates and the skipping proportions are significantly higher in Task 3, the only task-specific paradigm. Because readers in Task 3 were searching for a particular relation, this does not necessarily require reading all sentences until the end; however, Fig. 5 shows that the probability of a word being skipped decreases for longer words, but short words are frequently skipped consistently in all tasks.

### Reading time

**Figure 6:**
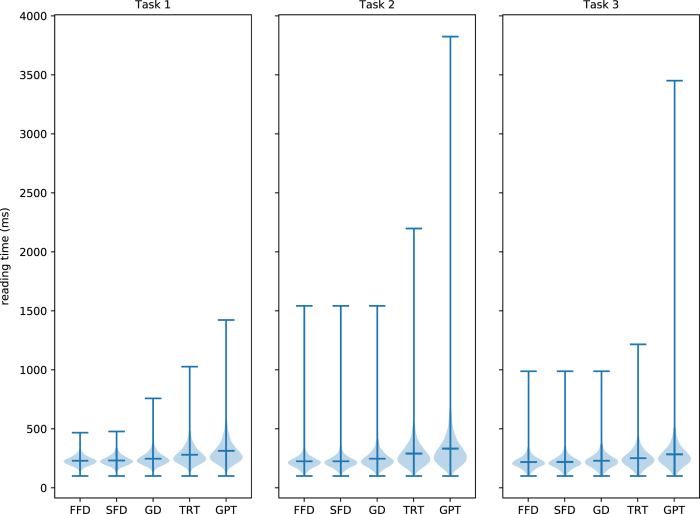
Violin plot showing means, distributions, and ranges of the reading time measures per word for each task and each eye-tracking feature (x-axis) in milliseconds.

The reading times in the recorded data were also validated. As described previously, we extracted the features described in the GECO corpus^5^: first fixation duration (FFD), single fixation duration (SFD), gaze duration (GD), total reading time (TRT) and go-past time (GPT). This is visualized in Fig. 6, which shows the mean and distributions of the reading times for each feature, separated by task. For reference, Supplementary Table S1 presents the exact values. For the calculation of these reading times only fixations >100 ms were considered. The presented ranges are in line with those presented by the previous study^5^.

### EEG

The aim of the technical validation of the EEG data was to replicate findings of previous studies investigating co-registration of EEG and eye-movement data during free reading tasks^12,21^.

Preprocessing of the EEG data was analogous to the feature extraction; however, after preprocessing a single bandpass filter was applied in addition (0.5–30 Hz). Afterwards, EEG data were re-referenced to the average reference and segmented to onsets of fixations. Analogous to Dimigen *et al.*^12^, a time-window of 1600 ms was chosen (600 ms before onset of the fixation to 1000 ms after). Furthermore, the pool of fixations was limited to first-pass reading fixations, which yielded in 154,173 trials in total.

### Fixation-related potentials

**Figure 7:**
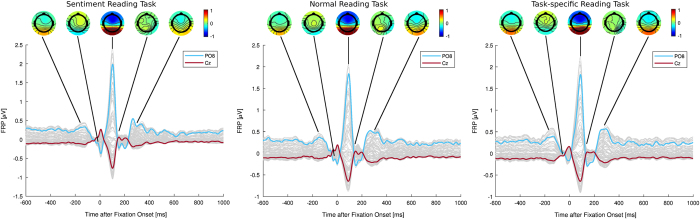
FRPs during the different task conditions with selected scalp level potential distributions. Topographies show amplitudes in microvolt, coded as color.

As a first validation step, fixation-related potentials (FRPs) were extracted. Therefore, the pool of fixations described above was divided into the three task conditions (Task 1, n = 56743; Task 2, n = 48723; Task 3, n = 48707) and trials were averaged within each condition. Data were baseline corrected using a 100 ms time-window before fixation onset. Figure 7 shows the time-series of the resulting FRPs for two electrodes (PO8 and Cz) as well as topographies of the voltage scalp distributions at selected timepoints. Due to the different EEG system in the study of Dimigen *et al.*^12^, electrode locations did not match perfectly^22^. In order to compare the results, we used similar electrodes. The time-points were chosen according to Dimigen *et al.*^12^, namely the visually evoked lambda response of the previous fixation (1), the mygoenic spike potential at saccade onset (2), the lambda response of the current fixation (3), the N170 component (4) and the N400 component, which is overlapped by the lambda response of the succeeding fixation (5).

All results are in line with the findings of Dimigen *et al.*^12^. The five ERP components (for which the scalp topographies are plotted) are highly similar to this study in the time-course of the chosen electrodes. This also applies for the scalp level topographies.

### Fixation duration effect on ERPs

**Figure 8:**
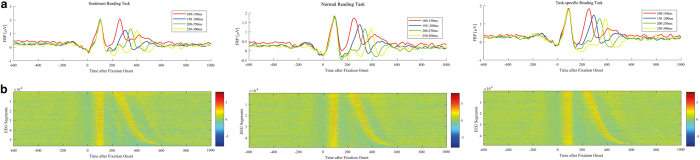
Clustered EEG segments. (**a**) FRPs of electrode Cz, clustered by duration of the fixation. (**b**) Each horizontal line represents the mean of the current and 50 adjacent EEG epochs, segmented on fixation onset. Segments are ordered by fixation duration (top: shortest fixation, bottom: longest fixation). Color represents the amplitude of the signal in microvolt.

Previous studies^12,22^ were able to show an effect of fixation duration on the resulting FRPs. We followed two approaches to demonstrate this dependency in the current dataset. The first followed the procedure of Dimigen *et al.*^12^, therefore all single-trial FRPs were ordered by fixation duration. As a next step, a vertical sliding time-window was used to smooth the data; that is, for each EEG segment, the average of 50 adjacent trials was calculated. Figure 8b shows the resulting plots per task condition. In line with this previous study^12^ a first positivation P1 can be identified at 100 ms post-fixation onset. A second positive peak P2 is located dependent on the duration of the fixation, which can be explained by the time-jittered succeeding fixation.

The second approach is based on previous related work^23^ in which single trial EEG segments are clustered by the duration of the current fixation. Here, four clusters were chosen (100–150 ms, 150–200 ms, 200–250 ms, 250–300 ms). Data within each cluster were averaged in order to get four different FRPs, depending on the fixation duration. The results can be seen in Fig. 8a. While P1 is located around 100 ms post-fixation onset, the P2 component also moves as a function of the fixation duration, which is consistent with the findings reported by Henderson *et al.*^22^.

## Additional information

**How to cite this article**: Hollenstein, N. *et al*. ZuCo, a simultaneous EEG and eye-tracking resource for natural sentence reading. *Sci. Data*. 5:180291 doi: 10.1038/sdata.2018.291 (2018).

**Publisher’s note**: Springer Nature remains neutral with regard to jurisdictional claims in published maps and institutional affiliations.

## Supplementary Material



Supplementary Table S1

## Figures and Tables

**Table 1:** Schematic overview of the three tasks in the study design.

	Task 1 Normal reading (Sentiment)	Task 2 Normal reading (Wikipedia)	Task 3 Task-specific reading (Wikipedia)
**Material**	Positive, negative or neutral sentences from movie reviews	Wikipedia sentences containing specific relations	Wikipedia sentences containing specific relations
**Example**	“*The film often achieves a mesmerizing poetry*.” (positive)	*“Talia Shire (born April 25, 1946) is an American actress of Italian descent.”* (relations: *nationality, job title*)	“*Lincoln was the first Republican president.*” (relation: *political affiliation*)
**Task**	Read the sentences, rating the quality of the movie based on the sentence read	Read the sentences, answer control questions	Mark whether a specific relation occurs in the given sentence or not
**Control question**	*“Based on the previous sentence, how would you rate this movie from 1 (very bad) to 5 (very good)?”*	*“Talia Shire was a …1) singer 2) actress 3) director”*	*“Does this sentence contain the* political affiliation *relation? 1) Yes 2) No”*

**Table 2:** Details of all subjects in the study; reading speed is measured in seconds (with standard deviation in brackets).

Subject ID	Age	Gender	LexTale	Reading speed Task 1	Reading speed Task 2	Reading speed Task 3	Score Task 1	Score Task 2	Score Task 3
ZKW	25	female	96.25%	6.94 (4.07)	11.73 (7.22)	6.14 (4.17)	69.57%	91.67%	94.84%
ZDN	32	male	97.50%	3.91 (1.49)	4.10 (1.62)	2.93 (1.61)	89.13%	86.11%	92.87%
ZPH	26	male	97.50%	4.78 (2.28)	7.55 (3.47)	2.71 (2.32)	89.13%	94.44%	97.05%
ZMG	51	male	100.00%	4.39 (2.47)	5.33 (3.25)	3.73 (2.77)	91.30%	88.89%	95.82%
ZAB	41	female	100.00%	4.88 (2.08)	5.14 (2.49)	3.32 (2.17)	76.09%	86.11%	90.42%
ZJN	51	female	97.50%	8.71 (4.63)	11.30 (6.55)	7.10 (5.05)	54.34%	83.33%	79.12%
ZKH	41	female	81.25%	5.42 (2.34)	6.43 (3.82)	5.57 (3.10)	76.09%	83.33%	93.12%
ZGW	49	male	91.25%	6.87 (3.58)	8.06 (4.31)	4.17 (3.01)	71.74%	86.11%	92.14%
ZJS	42	male	97.50%	4.34 (1.95)	4.18 (2.09)	2.88 (1.71)	91.30%	91.67%	93.86%
ZKB	26	female	100.00%	5.39 (2.96)	8.43 (4.74)	2.48 (1.63)	89.13%	86.11%	95.33%
ZDM	25	male	100.00%	4.41 (2.23)	5.13 (2.42)	3.32 (2.20)	76.09%	80.56%	96.81%
ZJM	41	male	77.50%	6.22 (3.17)	8.73 (5.07)	6.30 (3.72)	80.43%	97.22%	96.56%
**average**	**38**	-	**94.69%**	**5.52**	**7.18**	**4.22**	**79.53%**	**87.96%**	**93.16%**

**Table 3:** Descriptive statistics of reading materials (M = mean, SD = standard deviation, R = range).

	Task 1 Normal reading (Sentiment)			Task 2 Normal reading (Wikipedia)			Task 3 Task-specific reading (Wikipedia)			Total		
**words**	7079			6386			8164			21629		
**word types**	3080			2657			2995			7099		
**sentences**	400			300			407			1107		
	**M**	**SD**	**R**	**M**	**SD**	**R**	**M**	**SD**	**R**	**M**	**SD**	**R**
**words per sentence**	17.70	8.29	3–43	21.29	10.55	5–62	20.06	10.09	5–62	19.54	9.72	3–62
**word length**	6.97	2.71	1–26	6.70	2.65	1–29	6.69	2.58	1–21	6.79	2.65	1–29
