# Supplementary material for: ZuCo, a simultaneous EEG and eye-tracking resource for natural sentence reading
Source: Sci Data. 2018 Dec 11;5:180291. doi: 10.1038/sdata.2018.291 (PMC6289117; doi:10.1038/sdata.2018.291)
Supplement: Supplementary Table S1 [file sdata2018291-s2.docx]

*Supplementary Table 1: mean (M), standard deviations (SD), and ranges of the
reading time measures per feature for each task in milliseconds*

| **Feature** | **Task 1** |  |  |  | **Task 2** |  |  |  | **Task 3** |  |  |  |
| --- | --- | --- | --- | --- | --- | --- | --- | --- | --- | --- | --- | --- |
|  | M | SD | Min | Max | M | SD | Min | Max | M | SD | Min | Max |
| **FFD** | 228.96 | 49.10 | 100 | 984.00 | 222.82 | 60.70 | 100 | 1542 | 219.08 | 57.24 | 100 | 988 |
| **SFD** | 235.42 | 59.27 | 100 | 984.00 | 226.76 | 69.81 | 100 | 1542 | 222.08 | 61.72 | 100 | 916 |
| **GD** | 262.73 | 84.42 | 100 | 1287.45 | 267.31 | 109.71 | 100 | 1880 | 242.78 | 80.27 | 100 | 1206 |
| **TRT** | 358.90 | 167.99 | 100 | 1839.00 | 370.55 | 199.01 | 100 | 3313 | 306.11 | 145.84 | 100 | 2100 |
| **GPT** | 444.79 | 362.52 | 100 | 5290.73 | 452.20 | 418.62 | 100 | 6935.33 | 398.81 | 383.43 | 100 | 13128 |
